# Associations of Individual and Neighborhood Factors with Disparities in COVID-19 Incidence and Outcomes

**DOI:** 10.5811/westjem.18526

**Published:** 2025-01-15

**Authors:** Margaret E. Samuels-Kalow, Rebecca E. Cash, Kori S. Zachrison, Auriole Corel Rodney Fassinou, Norman Harris, Carlos A. Camargo

**Affiliations:** * Harvard Medical School, Massachusetts General Hospital, Department of Emergency Medicine, Boston, Massachusetts; † Cornell University, Ithaca, New York; ‡ Temple University, Lewis Katz School of Medicine, Philadelphia, Pennsylvania

## Abstract

**Introduction:**

The disproportionate impact of coronavirus 2019 (COVID-19) on Black and Hispanic communities has been widely reported. Many studies have used neighborhood racial/ethnic composition to study such disparities, but less is known about the interplay between individual race/ethnicity and neighborhood racial composition. Therefore, our goal in this study was to assess the relative contributions of individual and neighborhood risk to disparities in COVID-19 incidence and outcomes.

**Methods:**

We performed a cross-sectional study of patients with emergency department (ED) and inpatient visits to an academic health system (12 hospitals; February 1–July 15, 2020). The primary independent variable was race/ethnicity; covariates included individual age, sex, comorbidity, insurance and neighborhood density, poverty, racial/ethnic composition, education and occupation. The primary outcome was severe acute respiratory syndrome coronavirus 2 (SARS-CoV-2) positivity; secondary outcomes included admission and death after COVID-19. We used generalized estimating equations to assess whether race/ethnicity remained significantly associated with COVID-19 after adjustment for individual and neighborhood factors.

**Results:**

There were 144,982 patients; 5,633 (4%) were SARS-CoV-2 positive. Of those, 2,961 (53%) were admitted and 601(11%) died. Diagnosis of COVID-19, admission, and death were more common among non-Hispanic Black, Hispanic, Spanish-speaking patients, and those with public insurance. In the base model (adjusting for race/ethnicity, age, sex, and comorbidities), race/ethnicity was strongly associated with COVID-19 (non-Hispanic Black odds ratio [OR] 4.64 [95% confidence interval (CI) 4.18–5.14], and Hispanic OR 6.99 [CI 6.21–7.86]), which was slightly attenuated but remained significant after adjustment for neighborhood factors. Among patients with COVID-19, there was no significant association between race/ethnicity and hospital admission, other than for patients with unknown race.

**Conclusion:**

This data demonstrates a persistent association between race/ethnicity and COVID-19 incidence, with Black and Hispanic patients at significantly higher risk, which was not explained by measured individual or neighborhood factors. This suggests that using existing neighborhood factors in studies examining health equity may be insufficient, and more work is needed to quantify and address structural factors and social determinants of health to improve equity.

Population Health Research Capsule What do we already know about this issue?

*The disproportionate impact of COVID-19 on Black and Hispanic communities has been widely reported.*

What was the research question?

*We sought to assess the relative contributions of individual and neighborhood risk to disparities in COVID-19 incidence and outcomes.*

What was the major finding of the study?

*Race/ethnicity was associated with COVID-19 and remained significant after adjustment for neighborhood factors.*

How does this improve population health?

*This data suggests that using existing neighborhood factors in studies examining health equity may be insufficient.*

## INTRODUCTION

The disproportionate impact of coronavirus 2019 (COVID-19) on Black and Hispanic communities has been extensive.^1^ ^–^ ^9^ Along with the association with individual race and ethnicity, multiple studies have shown associations between neighborhood demographics and COVID-19 incidence and outcomes. Areas with higher proportions of Black and Hispanic residents have higher COVID-19 incidence and fatality rates.^10^ ^–^ ^13^ Although a number of other neighborhood variables have been associated with increased rates of COVID-19, including poverty, insurance coverage,^13^ unemployment, essential service employment,^14^ and neighborhood education levels,^11^ the association with neighborhood demographic composition may be stronger than that with neighborhood socioeconomic status (SES).^15^ Importantly, the association between community-level social determinants of health, such as neighborhood poverty and COVID-19 rates, does not seem to be explained by differential testing rates.^14^

The connection between social determinants of health, unequal exposure to severe acute respiratory syndrome coronavirus 2 (SARS-CoV-2), and race/ethnicity is a reflection of structural racism^16^—the “discriminatory policies, practices, and systems that reinforce an unequal distribution of power and resources in social institutions.”^17^ Non-White patients have increased representation in service-industry or low-income jobs, increased financial insecurity, more frequent residence in shared or congregate housing,^18^ and a resulting increased risk of exposure to COVID-19. In addition, structural racism also affects patients’ lived experience in the community and healthcare settings, and specifically their ability to access care.^19^ Our previous work using data from a large academic health system has demonstrated co-location (overlap) of neighborhood factors, such as percentage of the population that was Hispanic, non-Hispanic Black, without health insurance or living in poverty, and COVID-19 cases.^13^ However, recent research has demonstrated that relying on neighborhood-level social risk factors alone may over-attribute findings to the neighborhood-level factor.^20^ Therefore, our goal in this study was to assess the relative contributions of individual and neighborhood risk to those disparities in a dataset where we could assess individual demographics, insurance status (as a proxy for individual SES), and comorbidities.

## METHODS

### Study Design and Population

We performed a cross-sectional study of all emergency department (ED) and inpatient visits from February 1–July 15, 2020 within a large academic health system of 12 hospitals, including two academic medical centers, in New England. Data were abstracted from the electronic health record (EHR). Visits were eligible for inclusion if they were either (1) the first encounter (ED visit or admission) to any health system facility during the study period where the patient was SARS-CoV-2 positive or (2) the first visit if the patient was never SARS-CoV-2 positive. Patients were eligible if they were a Massachusetts state resident based on recorded address. We excluded patients if their address was a PO box or if they were undomiciled or not able to be geocoded via Epic or ArcGIS (Environmental Systems Research Institute, Inc, Redlands, CA) (for a total of 2,233 or 2% of participants; see eFigure1). Each patient was included only once.

#### Ethics approval

All methods were carried out in accordance with institutional guidelines; in particular, the study was deemed exempt from informed consent requirements by the Mass General Brigham Institutional Review Board. Because of this, the datasets generated and analyzed during the current study are not publicly available due to patient privacy concerns and data confidentiality rules.

### Variable Definitions

The primary predictor variable was race/ethnicity. For a subset of 2,494 patients in the health system, Hispanic/Latino was included as a race. Patients were considered to be Hispanic if their race was Hispanic/ Latino, if their ethnicity was Hispanic/Latino regardless of race, or if their ethnicity was “Brazilian,” “Dominican,” “Honduran,” “Puerto Rican,” “Salvadoran,” “Guatemalan,” “Columbian,” or “Mexican, Mexican American, Chicano,” regardless of race. Patients were otherwise categorized based on reported race and categorized as non-Hispanic White, non-Hispanic Black, Asian, other, missing, or unknown.

Sex was defined as male or female; data on eight patients whose sex assignment at birth was not known was coded as “missing.” Similarly, data on 22 patients whose reported age was >107 years was also recorded as “missing” as it was presumed to represent an error in the EHR. Given the limited data on individual social determinants of health in the EHR, we chose to use insurance status and language as factors that are associated with access and utilization of care generally^21^ ^,^ ^22^ and, specifically, for COVID-19.^13^ ^,^ ^23^ Insurance status was defined as public, private or other (see Appendix), and preferred language abstracted from the EHR was English, Spanish, Portuguese, Creole-Haitian, Arabic, other, or missing/unknown (null, declined, unavailable).

We ascertained comorbidities by calculation of the Charlson Comorbidity Index (CCI) calculated from International Classification of Diseases, 10^th^ Rev, Clinical Modification codes in the problem list,^24^ ^,^ ^25^ and by direct identification of obesity, pulmonary disease, chronic kidney disease, diabetes mellitus, and hypertension in the problem list (see Appendix). Comorbidities were chosen based on previously published associations^2^ ^,^ ^26^ ^,^ ^27^ and institutional guidance regarding risk factors for poor outcomes from COVID-19.

Neighborhood factors to assess social determinants of health were drawn from the 2015–2019 American Community Survey five-year estimates^28^ and included neighborhood poverty,^13^ educational attainment, service occupation,^29^ and population density^30^ as well as proportion Hispanic/Latino, non-Hispanic Black, non-Hispanic Asian, bachelor’s degree or higher, and working in service occupation analyzed at the census tract level. We derived quintiles for neighborhood factors for analysis using all census tracts in the state of Massachusetts rather than those included in the sample.

### Outcomes

The primary outcome was SARS-CoV-2 positivity (COVID-19 positivity), defined as polymerase chain reaction test result of positive or admission/discharge status documented as “COVID-19 Positive” or “Patient Expired (COVID-19).” Secondary outcomes were hospital admission after SARS-CoV-2 positivity (admission after COVID-19), defined as admission during first encounter where SARS-CoV-2 positive or within 14 days of the first encounter; or death after SARS-CoV-2 positivity (death after COVID-19), defined as death at any time during the study period after the first encounter where the SARS-CoV-2 test result was positive. Outcomes were ascertained from the EHR.

### Statistical Analysis

We performed all statistical analyses with Stata SE 15.1 (StataCorp, College Station, TX), with *P* < 0.05 considered statistically significant. Continuous variables are displayed as mean (SD). Categorical variables are displayed as numbers (percentage) of participants within each group. Participants with missing data were excluded from models. We calculated descriptive statistics for patients and neighborhood characteristics in the sample overall and by COVID-19 outcomes.

#### Association between race/ethnicity and individual-level risk factors

We used multivariable logistic regression models to assess the association between comorbidities and individual risk factors and race/ethnicity. Given that we hypothesized that comorbidities were likely on the causal pathway between race/ethnicity and increased COVID-19 burden of disease, we also assessed the individual comorbidities as mediators of the association between race/ethnicity and COVID-19 incidence and outcomes. We used the Baron and Kenny methodology to assess for mediation.^31^ First, we confirmed a significant association between race/ethnicity and each outcome (COVID-19 positivity, admission, and death) and between race/ethnicity and each mediator. Next, we included each mediator in a model with race/ethnicity and the outcome. We assessed for any major change in estimates, in particular a change in magnitude, direction, or statistical significance. Complete mediation would be indicated by the association between race/ethnicity and the outcome becoming non-significant once the mediator was included.

#### Accounting for neighborhood-level factors

Once we had determined that there were no significant mediation effects, we assessed neighborhood factors for multicollinearity using variance inflation factors (VIF) in a linear regression model. A VIF greater than 10 was considered indication of potential multicollinearity. We used generalized estimating equations to assess whether race/ethnicity remained significantly associated with COVID-19 positivity after adjustment for neighborhood factors and insurance status. We used a logit link, binomial distribution, and working independence correlation structure to estimate odds ratios separately for each outcome (COVID-19 positivity, admission, and death) with robust standard errors and clustering at the neighborhood level. For each outcome, a base model was created that included age, sex, and CCI. We chose to use the CCI as a summary measure of overall comorbidity status. We then generated models including each neighborhood factor individually and a fully adjusted model.

#### Sensitivity analyses

We conducted sensitivity analyses stratified by comorbidity and including insurance status in the base model. The analysis stratified by comorbidities was designed to address the concern that the distribution of comorbidities in our population would be unequal by race^32^ ^,^ ^33^ and, therefore, adjusting for comorbidities might diminish the overall impact of structural racism and disadvantage. The inclusion of insurance was designed to assess the impact of including a proxy measure of individual SES in the model.

## RESULTS

There were 144,982 patients with ED visits or hospital admissions during the study period of whom 5,633 (4%) were COVID-19 positive (Table 1). Of those, 2,961 (53%) were admitted and 601 (11%) died. Although non-Hispanic Black patients were 9% of the overall cohort, they represented 16% of the COVID-19 positive patients, 17% of the admitted patients, and 15% of the patients who died after testing positive for COVID-19.

**Table 1.** Patient characteristics overall and by COVID-19 outcomes.

			Among COVID-19 positive	
Patient characteristic	Overall	COVID-19 positive	Admitted	Died
Overall, N (%)	144,982 (100)	5,633 (4)	2,961 (53)	601 (11)
Race/ethnicity, n (%)				
Non-Hispanic White	90,605 (62)	1,979 (35)	1,310 (44)	377 (63)
Non-Hispanic Black	12,824 (9)	894 (16)	489 (17)	90 (15)
Hispanic/Latino	19,115 (13)	1,667 (30)	692 (23)	63 (10)
Asian	5,496 (4)	192 (3)	102 (3)	13 (2)
Other	7,539 (5)	541 (10)	226 (8)	29 (5)
Missing or unknown	9,403 (6)	360 (6)	142 (5)	29 (5)
Age, mean (SD), years	44.6 (24.5)	54.3 (20.5)	63.0 (18.9)	75.7 (13.9)
Missing	22	0	0	0
Sex, n (%)				
Female	79,550 (55)	2,717 (48)	1,388 (47)	253 (42)
Male	65,424 (45)	2,916 (52)	1,573 (53)	348 (58)
Comorbidities, n (%)				
Charlson comorbidity index, mean (SD)	0.9 (1.6)	1.0 (1.7)	1.4 (1.9)	2.4 (2.4)
Obesity	15,473 (13)	827 (17)	492 (17)	84 (14)
Pulmonary disease	36,042 (31)	2,113 (43)	1,487 (50)	386 (65)
Chronic kidney disease	8,263 (7)	612 (13)	503 (17)	189 (32)
Diabetes mellitus	14,230 (12)	1,211 (25)	910 (31)	216 (36)
Hypertension	37,306 (32)	2,076 (42)	1,502 (51)	390 (65)
Missing comorbidities	27,389	738	8	4
Insurance, n (%)				
Private only	78,939 (55)	2,183 (39)	1,085 (37)	179 (30)
Public only	36,384 (25)	2,232 (39)	1,188 (40)	223 (37)
Other or multiple	27,489 (19)	1,174 (21)	673 (23)	199 (33)
Any public insurance, n (%)	60,515 (42)	3,054 (55)	1,787 (61)	418 (70)
Missing	2,170	44	15	0
Language preference, n (%)				
English	124,541 (86)	3,348 (59)	1,896 (64)	443 (74)
Spanish	12,879 (9)	1,695 (30)	737 (25)	74 (12)
Portuguese	991 (1)	50 (1)	18 (1)	4 (1)
Haitian Creole	543 (0)	124 (2)	89 (3)	24 (4)
Arabic	458 (0)	13 (0)	9 (0)	42 (7)
Other	3,563 (2)	242 (4)	154 (5)	14 (2)
Missing or unknown	2,007 (1)	161 (3)	58 (2)	443 (74)

*COVID-19*, coronavirus 2019.

Similarly, Hispanic patients were 13% of the overall cohort, 30% of the COVID-19 positive patients, 23% of the admitted patients and 10% of the patients who died with COVID-19. Patients who were listed as having a language preference of Spanish were 9% of the overall cohort but 30% of the COVID-19 positive patients, 25% of the admitted patients, and 12% of those who died with COVID-19. Although 36,384 patients (25%) in the overall population had only public insurance, 39% of the COVID-19 positive, 40% of the patients admitted after COVID-19, and 37% of the patients who died after COVID-19 had only public insurance. When insurance was examined as those who had any public insurance (60,515, 42%), there were higher percentages of patients with public insurance who were COVID-19 positive (55%), admitted after COVID-19 (61%), or died after COVID-19 (70%) than in the overall cohort.

Regarding neighborhood characteristics (Table 2), there was a greater representation of patients from neighborhoods in the highest quintile of poverty, percentage Hispanic population, percentage non-Hispanic Black population and percentage service occupation, and lowest quintile of non-Hispanic White population and educational attainment within the COVID-19 outcome groups (tested positive, admitted, died).

**Table 2.** Neighborhood characteristics overall and by COVID-19 outcomes.

			Among COVID-19 positive	
Neighborhood characteristic	Overall	COVID-19 positive	Admitted	Died
Density, mean (SD), population per square mile	10,032 (11984)	16,121 (13,592)	15,062 (13529)	12,402 (11711)
Families living below poverty, n (%)				
Lowest quintile (0–1.5%)	28,375 (20)	634 (11)	382 (13)	83 (14)
2 (1.6–3.4%)	29,941 (21)	655 (12)	391 (13)	95 (16)
3 (3.5–6.3%)	26,173 (18)	712 (13)	400 (14)	80 (13)
4 (6.4–13.5%)	32,572 (22)	1,736 (31)	902 (31)	191 (32)
Highest quintile (13.6–65.2%)	27,849 (19)	1,890 (34)	882 (30)	152 (25)
Missing	72	6	4	0
Hispanic/Latino population, n (%)				
Lowest quintile (0–2.2%)	21,932 (15)	363 (6)	211 (7)	63 (10)
2 (2.3–4.5%)	27,409 (19)	584 (10)	379 (13)	99 (16)
3 (4.6–8.3%)	28,998 (20)	698 (12)	430 (15)	82 (14)
4 (8.4–19.1%)	27,399 (19)	962 (17)	498 (17)	110 (18)
Highest quintile (19.2–100%)	39,202 (27)	3,024 (54)	1,443 (49)	247 (41)
Non-Hispanic Black population, n (%)				
Lowest quintile (0–0.6%)	21,829 (15)	474 (8)	292 (10)	62 (10)
2 (0.7–2.0%)	24,281 (17)	506 (9)	282 (10)	73 (12)
3 (2.1–4.2%)	34,356 (24)	1,363 (24)	816 (28)	180 (30)
4 (4.3–9.2%)	27,814 (19)	970 (17)	511 (17)	98 (16)
Highest quintile (9.3–82.9%)	36,660 (25)	2,318 (41)	1,060 (36)	188 (31)
Non-Hispanic Asian population, n (%)				
Lowest quintile (0–0.7%)	20,830 (14)	556 (10)	296 (10)	71 (12)
2 (0.8–2.2%)	22,773 (16)	786 (14)	422 (14)	106 (18)
3 (2.3–4.9%)	30,938 (21)	1,194 (21)	602 (20)	110 (18)
4 (5.0–10.9%)	37,281 (26)	1,869 (33)	972 (33)	171 (28)
Highest quintile (11.0–59.2%)	33,118 (23)	1,226 (22)	669 (23)	143 (24)
Non-Hispanic White population, n (%)				
Lowest quintile (0–50.2%)	38,229 (26)	2,977 (53)	1,384 (47)	227 (38)
2 (50.3–72.3%)	28,958 (20)	1,098 (20)	606 (20)	129 (21)
3 (72.4–83.7%)	31,217 (22)	742 (13)	464 (16)	105 (17)
4 (83.8–91.4%)	28,150 (19)	574 (10)	366 (12)	98 (16)
Highest quintile (91.5–100%)	18,386 (13)	240 (4)	141 (5)	42 (7)
Bachelor’s degree or higher level of education, n (%)				
Lowest quintile (0–22.3%)	24,773 (17)	1,926 (34)	878 (30)	136 (23)
2 (22.4–34.4%)	21,154 (15)	1,097 (19)	578 (20)	106 (18)
3 (34.5–46.5%)	27,471 (19)	756 (13)	406 (14)	101 (17)
4 (46.6–63.9%)	34,611 (24)	949 (17)	525 (18)	125 (21)
Highest quintile (64.0–95.6%)	36,930 (25)	903 (16)	574 (19)	133 (22)
Persons in service occupations, n (%)				
Lowest quintile (0–10.8%)	33,289 (23)	817 (15)	509 (17)	112 (19)
2 (10.9–14.8%)	31,728 (22)	723 (13)	430 (15)	111 (18)
3 (14.9–18.9%)	23,812 (16)	565 (10)	316 (11)	80 (13)
4 (19.0–25.2%)	23,950 (17)	1,028 (18)	508 (17)	116 (19)
Highest quintile (25.3–69.2%)	32,161 (22)	2,498 (44)	1,198 (40)	182 (30)

*COVID-19*, coronavirus 2019.

### Collinearity

For the outcome of COVID-19 positivity, we detected multicollinearity when including patient race/ethnicity and all neighborhood race/ethnicity variables; excluding variable for quintiles of non-Hispanic White population resolved the collinearity issues. For the outcomes of admission within 14 days of first COVID-19 positive presentation and death any time after first COVID -19 positive presentation (among COVID-19 positive patients), multicollinearity was detected between race/ethnicity variables and service occupation. Removing the variables for quintiles of non-Hispanic White population and service occupation resolved the collinearity.

### Individual Comorbidity and Risk Factors: Association and Mediation

There were significant associations between race/ethnicity and each comorbidity, with the exception of non-Hispanic Black and hypertension (HTN) (eTable 1). In the unadjusted model for COVID-19 positivity (Table 3), race/ethnicity was a significant predictor of COVID-19 positivity (non-Hispanic Black odds ratio [OR] 3.36 [3.09–3.64] and Hispanic OR 4.28 [4.00–4.58]). The association with race/ethnicity remained significant even after adjustment for each individual comorbidity and risk factor (Table 3). There was potential partial mediation by language preference (Table 3), as the OR was substantially decreased but still statistically significant after adjustment for language preference.

**Table 3.** Association of race/ethnicity and COVID-19 positivity^a^, unadjusted and adjusted for each comorbidity and individual risk factor.

	Race/ethnicity (primary exposure), OR (95% CI)^b^					
	Non-Hispanic White	Non-Hispanic Black	Hispanic/ Latino	Asian	Other	Missing or unknown
Unadjusted model	1.00 (referent)	3.36 (3.09–3.64)	4.28 (4.00–4.58)	1.62 (1.39–1.88)	3.46 (3.14–3.82)	1.78 (1.59–2.00)
Adjusted for each comorbidity						
CCI	1.00 (referent)	3.61 (3.31–3.93)	4.57 (4.25–4.92)	1.89 (1.61–2.22)	4.01 (3.60–4.47)	1.83 (1.61–2.08)
Obesity	1.00 (referent)	3.50 (3.21–3.81)	4.26 (3.96–4.58)	1.83 (1.56–2.15)	3.74 (3.36–4.16)	1.71 (1.50–1.94)
Pulmonary disease	1.00 (referent)	3.65 (3.34–3.97)	4.47 (4.16–4.81)	1.96 (1.67–2.30)	3.95 (3.55–4.40)	1.99 (1.75–2.26)
Chronic kidney disease	1.00 (referent)	3.49 (3.21–3.81)	4.51 (4.19–4.85)	1.88 (1.60–2.21)	3.98 (3.57–4.44)	1.79 (1.58–2.04)
Diabetes mellitus	1.00 (referent)	3.33 (3.05–3.63)	4.23 (3.93–4.55)	1.84 (1.57–2.16)	3.90 (3.50–4.35)	1.83 (1.61–2.08)
Hypertension	1.00 (referent)	3.57 (3.28–3.90)	4.80 (4.46–5.17)	2.02 (1.72–2.37)	4.33 (3.88–4.83)	2.06 (1.81–2.34)
Adjusted for each individual risk factor						
Insurance type	1.00 (referent)	3.33 (3.06–3.62)	3.99 (3.72–4.29)	1.79 (1.53–2.08)	3.43 (3.11–3.80)	1.88 (1.67–2.11)
Any public Insurance	1.00 (referent)	3.33 (3.07–3.61)	4.04 (3.77–4.32)	1.72 (1.47–2.00)	3.44 (3.11–3.79)	1.83 (1.63–2.05)
Language preference	1.00 (referent)	2.97 (2.73–3.23)	1.78 (1.62–1.96)	1.33 (1.14–1.56)	1.98 (1.78–2.21)	1.22 (1.08–1.38)

a COVID-19 positivity was defined as PCR test result of positive or admission/discharge status documented as “COVID-19 Positive” or “Patient Expired (COVID-19).”

b Presented are odds ratios (95% CI) for the outcome (COVID-19 positivity) for each race/ethnicity group compared to non-Hispanic Whites (referent, first column), with the unadjusted values in the first row and after adjustment for the variables separately in following rows.

*CCI*, Charlson Comorbidity Index; *COVID-19*, coronavirus 2019.

In the unadjusted model for admission among the patients with COVID-19, non-Hispanic Black and Hispanic patients had lower odds of being admitted (OR 0.62 [0.52–0.72] and 0.36 [0.32–0.41], respectively), and the directionality and significance of the association was not altered by adjustment for any of the individual comorbidities or risk factors (eTables 2 and 3); a similar pattern was seen for deaths after COVID-19 (eTables 4 and 5). Together, this data suggests that the individual comorbidities and social risk factors are not significant mediators of the association between race/ethnicity and COVID-19 incidence and outcomes.

### Full Model: Race/Ethnicity, Individual and Neighborhood Factors, and COVID-19

Once there was less concern for comorbidities serving as a mediator of the association, we created a base model that included age, sex, and the CCI to examine how the associations between race/ethnicity and COVID-19 outcomes would change with the inclusion of neighborhood factors. In the base model (Table 4), race/ethnicity was strongly associated with COVID-19 positivity (non-Hispanic Black, OR 4.64 [4.18–5.14], Hispanic, OR 6.99 [6.21–7.86], which was slightly attenuated but remained significant after adjustment for neighborhood factors (non-Hispanic Black, OR 3.27 [2.90–3.69], Hispanic, OR 4.10 [3.66–4.60]]). Trends for other racial/ethnic groups are displayed in Table 4. Among patients with COVID-19, there was no significant association between race/ethnicity and hospital admission, other than that patients with missing or unknown race were less likely to be admitted, and that association remained consistent after adjustment for neighborhood factors. For the outcome of death after COVID-19, Hispanic (OR 0.62 [0.46–0.83]) and Asian (0.46 [0.25–0.86]) patients had lower odds of dying as compared to non-Hispanic White patients in the base model, and that association persisted after adjustment for neighborhood factors (Hispanic OR 0.61 [0.44–0.85], Asian OR 0.47 [0.25–0.91]) (Table 4).

**Table 4.** Odds ratios^a^ for the association between race/ethnicity and COVID-19 outcomes.

	COVID-19 positivity (N = 117,589; cluster n = 1,447)		Admission after COVID-19 (n = 4,895; cluster n = 848)		Death after COVID-19 (n = 4,895; cluster n = 848)	
	Base model	Fully adjusted^b^	Base model	Fully adjusted^c^	Base model	Fully adjusted^c^
Race/ethnicity						
Non-Hispanic White	1.00 (referent)	1.00 (referent)	1.00 (referent)	1.00 (referent)	1.00 (referent)	1.00 (referent)
Non-Hispanic Black	4.64 (4.18–5.14)	3.27 (2.90–3.69)	1.06 (0.87–1.30)	1.23 (0.99–1.54)	0.91 (0.69–1.20)	0.89 (0.66–1.21)
Hispanic/Latino	6.99 (6.21–7.86)	4.10 (3.66–4.60)	1.03 (0.87–1.22)	1.05 (0.86–1.29)	0.62 (0.46–0.83)	0.61 (0.44–0.85)
Asian	2.52 (2.08–3.04)	2.00 (1.66–2.41)	0.91 (0.66–1.25)	0.92 (0.65–1.30)	0.46 (0.25–0.86)	0.47 (0.25–0.91)
Other	5.94 (5.15–6.86)	3.80 (3.34–4.32)	0.88 (0.69–1.13)	0.88 (0.68–1.14)	0.80 (0.53–1.22)	0.79 (0.51–1.22)
Missing or unknown	3.66 (3.12–4.30)	3.18 (2.73–3.69)	0.60 (0.44–0.80)	0.62 (0.46–0.84)	0.75 (0.46–1.20)	0.74 (0.45–1.23)

a Estimated using generalized estimating equations (binomial distribution, logit link, working independence correlation structure) with robust standard errors and clustering at the neighborhood level. All models (base and fully adjusted) include race/ethnicity, age, sex, and the Charlson Comorbidity Index.

b Includes all neighborhood factors (density, poverty, Hispanic/Latino, Non-Hispanic Black, Non-Hispanic Asian, Bachelor’s degree or higher level of education and service occupation). Non-Hispanic White population within the census tract (neighborhood level variable) was excluded due to multicollinearity.

c Includes all neighborhood factors (density, poverty, Hispanic/Latino, Non-Hispanic Black, Non-Hispanic Asian, Bachelor’s degree or higher education level). Non-Hispanic White population within the census tract (neighborhood level variable) and service occupation excluded due to multicollinearity.

*COVID-19*, coronavirus 2019.

We further examined the association between race/ethnicity and COVID-19 outcomes in models stratified by comorbidity (eTables 6–10). For patients with and without obesity, race/ethnicity remained significantly associated with COVID-19 positivity in both the base and fully adjusted models, although the association was smaller in the obese patients for those who were Black or Hispanic and larger for those who were Asian. Similar trends were seen for pulmonary disease, although with smaller changes. For patients with chronic kidney disease, diabetes and HTN, the association with race/ethnicity was weaker in patients with the condition than those without, although it remained significant in all models.

As a sensitivity analysis, we modeled the association between race/ethnicity and COVID-19 including not only age, sex, and the CCI, but also insurance status as a marker of individual SES. Again, race remained strongly associated with COVID-19 positivity; only missing race was associated with admission for COVID-19, and Hispanic and Asian race/ethnicity were associated with lower odds of death after COVID-19 (Table 5).

**Table 5.** Odds ratios^a^ for the association between race/ethnicity and COVID-19 outcomes, including adjustment for insurance status.

	COVID-19 positivity (N = 116,631; cluster n = 1,447)		Admission after COVID-19 (n = 4,895; cluster n = 848)		Death after COVID-19 (n = 4,895; cluster n = 848)	
	Base model	Fully adjusted^b^	Base model	Fully adjusted^c^	Base model	Fully adjusted^c^
Race/ethnicity						
Non-Hispanic White	1.00 (referent)	1.00 (referent)	1.00 (referent)	1.00 (referent)	1.00 (referent)	1.00 (referent)
Non-Hispanic Black	4.22 (3.79–4.70)	3.16 (2.80–3.56)	1.06 (0.86–1.30)	1.25 (0.99–1.57)	0.89 (0.67–1.17)	0.88 (0.65–1.19)
Hispanic/Latino	5.97 (5.26–6.79)	3.83 (3.41–4.31)	0.98 (0.83–1.17)	1.02 (0.84–1.26)	0.60 (0.45–0.81)	0.60 (0.43–0.83)
Asian	2.44 (2.02–2.95)	1.97 (1.62–2.38)	0.90 (0.65–1.25)	0.93 (0.65–1.31)	0.47 (0.25–0.88)	0.49 (0.26–0.94)
Other	5.41 (4.67–6.29)	3.67 (3.22–4.19)	0.85 (0.66–1.10)	0.86 (0.66–1.13)	0.79 (0.52–1.21)	0.78 (0.51–1.21)
Missing or unknown	3.57 (3.04–4.19)	3.15 (2.71–3.66)	0.57 (0.43–0.77)	0.61 (0.45–0.82)	0.74 (0.46–1.18)	0.74 (0.45–1.22)

a Estimated using generalized estimating equations (binomial distribution, logit link, working independence correlation structure) with robust standard errors and clustering at the neighborhood level. All models (base and fully adjusted) include race/ethnicity, age, sex, the Charlson Comorbidity Index, and insurance status.

b Includes all neighborhood factors (density, poverty, Hispanic/Latino, Non-Hispanic Black, Non-Hispanic Asian, Bachelor’s degree or higher education level and service occupation). Non-Hispanic White population within the census tract (neighborhood level variable) was excluded due to multicollinearity.

c Includes all neighborhood factors (density, poverty, Hispanic/Latino, Non-Hispanic Black, Non-Hispanic Asian, Bachelor’s degree or higher education level). Non-Hispanic White population within the census tract (neighborhood level variable) and service occupation excluded due to multicollinearity.

*COVID-19*, coronavirus 2019.

## DISCUSSION

In this cross-sectional study of health system data from the initial stages of the COVID-19 pandemic, non-Hispanic Black, Hispanic, and Asian race/ethnicity were significantly associated with increased COVID-19 positivity, and the association remained significant after adjustment for both individual risk factors (age, sex, comorbidity, insurance) and neighborhood risk factors (density, poverty, racial/ethnic composition, educational attainment, occupation). These results demonstrate a persistent association with race/ethnicity after adjustment for potential explanatory factors (eg, comorbidities). Importantly, we did not find that comorbidities or individual insurance status (as a marker of SES) were meaningful mediators of the association between race/ethnicity and COVID-19 incidence or outcomes, meaning that we did not see evidence that they were on the causal pathway for this association. Additionally, the association was not fully explained by measured neighborhood risk.

From these results we draw two major conclusions. Firstly, recognizing that the residual association with measured race/ethnicity represents structural racism rather than biological variation and that there is no standard measurement for structural racism in administrative datasets, this data emphasizes the need for improved measurement of individual-level social determinants of health and the impact of structural racism. Similar to prior reports, we found stronger associations between race/ethnicity and COVID-19 positivity, again suggesting that higher rates in Black and Hispanic populations are driven by exposure,^4^ and that the mortality trends are more complex.^6^ ^,^ ^7^ This data builds upon prior reports that show a consistent impact of race and ethnicity that appears to be modified or mediated by social determinants of health.^26^ ^,^ ^30^ ^,^ ^34^ ^,^ ^35^ Similarly, a recent study demonstrated limited ability of insurance to correctly classify SES, as defined by education and income.^36^ Additional work is needed to define and reliably measure individual sociodemographic factors associated with disease vulnerability and use them to define areas for potential intervention.

The second major conclusion of our study urges caution in the use of neighborhood socioeconomic factors alone to examine disparities. Neighborhood factors represent the ecological exposure and not the individual experience, and this study demonstrates the complex interplay between these individual and neighborhood factors. For example, a study examining hospitalized patients with COVID-19 in Michigan found that those from socially vulnerable neighborhoods were more likely to present with severe disease, even after adjustment for age, sex, and comorbidities, but that neighborhood vulnerability was not associated with mortality.^37^ Overall, this data emphasizes the importance of measuring the factors (eg, individual housing insecurity, crowding, and essential occupations that could not be completed remotely) that reflect structural racism, and may serve as potential mediators in the association between race/ethnicity and COVID-19, rather than relying on neighborhood-level measurements alone. Future directions for this research could include using improved measurements of individual-level social determinants of health in future investigations in other conditions and interventions to reduce the disproportionate burden of disease.

## LIMITATIONS

Limitations of this study include that the data was drawn from a single health system and, therefore, may minimize hospital-level differences and disparities in care^38^ and may not fully capture the underlying population. This is particularly important because data from our city has shown differences in the racial/ethnic makeup of patient populations by hospital.^39^ ^,^ ^40^ However, the health system includes the hospital that has cared for the highest number of admitted COVID-19 patients in the area,^41^ ^,^ ^42^ and our prior work showed that our health system data identified similar clusters to the state data within our catchment area.^13^ Early in the pandemic there were disparities in testing access,^43^ ^,^ ^44^ although other studies in Massachusetts have found disparities that were not fully explained by testing access differences.^14^ Additionally, not all comorbidities may have been coded in the problem list, particularly for patients who were new to our health system, potentially limiting our ability to ascertain them. We used insurance status as a proxy for individual SES because we do not have full data on social determinants of health for all patients in the cohort.

It is challenging to determine whether the measured differences in admission and death were due to unmeasured differences in illness severity or comorbidities, represented ascertainment bias due to use of EHR data, or were a manifestation of implicit bias. Increased mortality has been reported in Hispanic populations,^8^ ^,^ ^9^ which was not demonstrated in our data, potentially reflecting unmeasured confounding from differences in the Hispanic population in our cohort (eg, healthy immigrant effect).^45^ Because we were interested in neighborhood effects, we were not able to include patients without an address, and there may be a different relationship between social risk and COVID-19 incidence and outcomes in an undomiciled population that we were unable to examine. Finally, with the ability of patients to access vaccination, and the evolution of new COVID-19 variants, the disparities in COVID-19 continue to evolve.

## CONCLUSION

The data shows a persistent association between non-Hispanic Black, Hispanic, and Asian race/ethnicity and higher COVID-19 incidence that is not explained by included individual or neighborhood factors. The results emphasize the importance of improving the measurement of structural factors and social determinants of health and careful attention to the use of individual-level and neighborhood-level risk factors in studies to enable interventions to improve the equity of pandemic response.

## Supplementary Information




