# Supplementary material for: Associations of Individual and Neighborhood Factors with Disparities in COVID-19 Incidence and Outcomes
Source: West J Emerg Med. 2025 Jan 15;26(2):315–25. doi: 10.5811/westjem.18526 (PMC11931697; doi:10.5811/westjem.18526)
Supplement: Supplementary file 1 [file wjem-26-315-s001.docx]

**Associations of individual and neighborhood factors with disparities in COVID-19 incidence and outcomes: Supplementary Tables**

Margaret E. Samuels-Kalow, MD, MPhil, MSHP;^1^ Rebecca E. Cash, PhD, MPH, NRP;^1^ Kori S. Zachrison MD MSc;^1^ Auriole Corel Rodney Fassinou;^2^ Norman Harris II BS’^3^

Carlos A. Camargo, Jr., MD, DrPH^1^

**Table of Contents:**

eFigure1. Flow chart of inclusion and exclusion criteria

eTable 1. Odds ratio (95% confidence interval) of each comorbidity for race

eTables 2-5. Association of race/ethnicity and COVID-19 outcomes of admission and death after COVID-19, unadjusted and adjusted for comorbidities and individual risk factors.

eTables 6-10. Odds ratios for the association between race/ethnicity and COVID-19 positivity stratified by comorbidities

**eFigure1. Flow chart of inclusion and exclusion criteria**

**
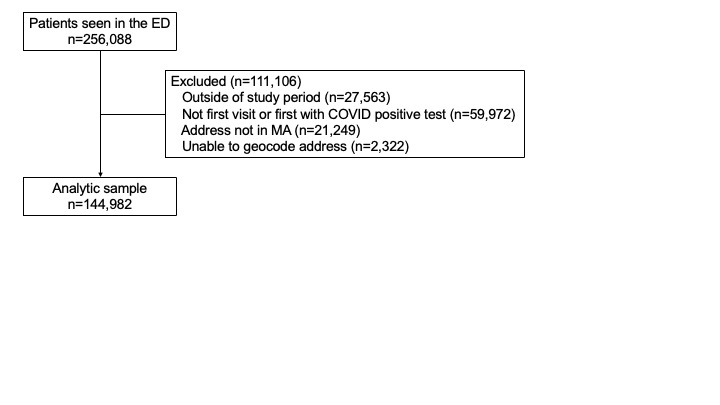
**

**eTable 1. Odds ratio (95% confidence interval) of each comorbidity for race/ethnicity** (among whole sample, n=144,982).

|  | **Obesity** | **Pulmonary disease** | **Chronic kidney disease** | **Diabetes Mellitus** | **Hypertension** |
| --- | --- | --- | --- | --- | --- |
| **Race/Ethnicity** |  |  |  |  |  |
| Non-Hispanic White | 1.00 (referent) | 1.00 (referent) | 1.00 (referent) | 1.00 (referent) | 1.00 (referent) |
| Non-Hispanic Black | 1.55 (1.47-1.64) | 0.85 (0.81-0.89) | 1.20 (1.12-1.29) | 1.62 (1.53-1.71) | 0.97 (0.93-1.01) |
| Hispanic/Latino | 1.66 (1.58-1.74) | 0.83 (0.80-0.86) | 0.53 (0.49-0.58) | 1.26 (1.20-1.33) | 0.52 (0.50-0.54) |
| Asian | 0.28 (0.24-0.33) | 0.57 (0.53-0.61) | 0.53 (0.46-0.62) | 0.87 (0.78-0.96) | 0.47 (0.44-0.51) |
| Other | 1.22 (1.13-1.32) | 0.74 (0.70-0.79) | 0.37 (0.32-0.43) | 0.76 (0.69-0.84) | 0.39 (0.36-0.42) |
| Missing or unknown | 0.30 (0.27-0.34) | 0.25 (0.23-0.27) | 0.28 (0.24-0.32) | 0.40 (0.36-0.44) | 0.20 (0.19-0.22) |

**eTables 2-5. Association of race/ethnicity and COVID-19 outcomes of admission and death after COVID-19, unadjusted and adjusted for comorbidities and individual risk factors.** These tables show the odds of the outcome for each race/ethnicity group compared to non-Hispanic Whites, with the unadjusted values in the first column and after adjustment for the variables in each individual column separately.

**eTable 2. Association of race/ethnicity and admission after COVID-19 ^a^, unadjusted and adjusted for each comorbidity.**

|  | **Unadjusted, OR (95% CI)** | **CCI** | **Obesity** | **Pulmonary disease** | **Chronic kidney disease** | **Diabetes Mellitus** | **HTN** |
| --- | --- | --- | --- | --- | --- | --- | --- |
| **Race/Ethnicity** |  |  |  |  |  |  |  |
| Non-Hispanic White | 1.00 (referent) | 1.00 (referent) | 1.00 (referent) | 1.00 (referent) | 1.00 (referent) | 1.00 (referent) | 1.00 (referent) |
| Non-Hispanic Black | 0.62 (0.52-0.72) | 0.80 (0.67-0.95) | 0.70 (0.59-0.83) | 0.75 (0.63-0.89) | 0.70 (0.59-0.83) | 0.66 (0.55-0.79) | 0.73 (0.61-0.87) |
| Hispanic/Latino | 0.36 (0.32-0.41) | 0.61 (0.52-0.71) | 0.47 (0.40-0.54) | 0.49 (0.43-0.57) | 0.51 (0.44-0.59) | 0.46 (0.40-0.54) | 0.55 (0.48-0.64) |
| Asian | 0.58 (0.43-0.78) | 0.76 (0.55-1.05) | 0.62 (0.45-0.85) | 0.67 (0.48-0.92) | 0.65 (0.47-0.90) | 0.61 (0.44-0.84) | 0.68 (0.49-0.94) |
| Other | 0.37 (0.30-0.45) | 0.60 (0.48-0.74) | 0.45 (0.36-0.56) | 0.47 (0.38-0.58) | 0.50 (0.40-0.62) | 0.47 (0.38-0.58) | 0.55 (0.44-0.68) |
| Missing or unknown | 0.33 (0.26-0.42) | 0.53 (0.41-0.68) | 0.40 (0.31-0.52) | 0.43 (0.33-0.55) | 0.44 (0.34-0.56) | 0.40 (0.31-0.52) | 0.47 (0.36-0.60) |

Abbreviations: CCI, Charlson comorbidity index.

^a^Admission after COVID was defined as admission during first encounter where COVID positive or within 14 days of first encounter.

**eTable 3. Association of race/ethnicity and admission after COVID-19^a^, unadjusted and adjusted for each individual risk factor.**

|  | **Unadjusted, OR (95% CI)** | **Insurance type** | **Any public insurance** | **Language preference** |
| --- | --- | --- | --- | --- |
| **Race/Ethnicity** |  |  |  |  |
| Non-Hispanic White | 1.00 (referent) | 1.00 (referent) | 1.00 (referent) | 1.00 (referent) |
| Non-Hispanic Black | 0.62 (0.52-0.72) | 0.63 (0.53-0.74) | 0.65 (0.55-0.77) | 0.56 (0.47-0.66) |
| Hispanic/Latino | 0.36 (0.32-0.41) | 0.35 (0.30-0.40) | 0.35 (0.30-0.40) | 0.33 (0.28-0.40) |
| Asian | 0.58 (0.43-0.78) | 0.61 (0.45-0.82) | 0.66 (0.49-0.89) | 0.49 (0.35-0.67) |
| Other | 0.37 (0.30-0.45) | 0.36 (0.30-0.44) | 0.37 (0.31-0.46) | 0.34 (0.28-0.42) |
| Missing or unknown | 0.33 (0.26-0.42) | 0.33 (0.26-0.41) | 0.34 (0.27-0.42) | 0.35 (0.27-0.45) |

^a^Admission after COVID was defined as admission during first encounter where COVID positive or within 14 days of first encounter.

**eTable 4. Association of race/ethnicity and death after COVID-19^a^, unadjusted and adjusted for each comorbidity.**

|  | **Unadjusted, OR (95% CI)** | **CCI** | **Obesity** | **Pulmonary disease** | **Chronic kidney disease** | **Diabetes Mellitus** | **HTN** |
| --- | --- | --- | --- | --- | --- | --- | --- |
| **Race/Ethnicity** |  |  |  |  |  |  |  |
| Non-Hispanic White | 1.00 (referent) | 1.00 (referent) | 1.00 (referent) | 1.00 (referent) | 1.00 (referent) | 1.00 (referent) | 1.00 (referent) |
| Non-Hispanic Black | 0.48 (0.37-0.61) | 0.56 (0.43-0.73) | 0.51 (0.39-0.65) | 0.55 (0.43-0.71) | 0.50 (0.39-0.65) | 0.48 (0.38-0.62) | 0.53 (0.41-0.68) |
| Hispanic/Latino | 0.17 (0.13-0.22) | 0.27 (0.21-0.36) | 0.20 (0.15-0.26) | 0.21 (0.16-0.28) | 0.23 (0.17-0.30) | 0.20 (0.15-0.26) | 0.24 (0.18-0.32) |
| Asian | 0.31 (0.17-0.55) | 0.42 (0.23-0.75) | 0.32 (0.18-0.57) | 0.35 (0.20-0.63) | 0.35 (0.19-0.62) | 0.32 (0.18-0.57) | 0.35 (0.20-0.63) |
| Other | 0.24 (0.16-0.36) | 0.40 (0.27-0.60) | 0.28 (0.19-0.42) | 0.30 (0.20-0.44) | 0.34 (0.23-0.50) | 0.29 (0.20-0.43) | 0.35 (0.24-0.52) |
| Missing or unknown | 0.37 (0.25-0.55) | 0.59 (0.39-0.90) | 0.40 (0.26-0.60) | 0.44 (0.29-0.67) | 0.46 (0.30-0.70) | 0.41 (0.27-0.62) | 0.48 (0.32-0.73) |

Abbreviations: CCI, Charlson comorbidity index.

^a^Death after COVID was defined as death anytime during study period after first encounter where COVID positive.

**eTable 5. Association of race/ethnicity and death after COVID-19^a^, unadjusted and adjusted for each individual risk factor.**

|  | **Unadjusted, OR (95% CI)** | **Insurance type** | **Any public insurance** | **Language preference** |
| --- | --- | --- | --- | --- |
| **Race/Ethnicity** |  |  |  |  |
| Non-Hispanic White | 1.00 (referent) | 1.00 (referent) | 1.00 (referent) | 1.00 (referent) |
| Non-Hispanic Black | 0.48 (0.37-0.61) | 0.51 (0.40-0.65) | 0.51 (0.40-0.65) | 0.40 (0.30-0.52) |
| Hispanic/Latino | 0.17 (0.13-0.22) | 0.16 (0.12-0.22) | 0.16 (0.12-0.21) | 0.19 (0.13-0.28) |
| Asian | 0.31 (0.17-0.55) | 0.37 (0.21-0.66) | 0.38 (0.21-0.68) | 0.22 (0.12-0.41) |
| Other | 0.24 (0.16-0.36) | 0.25 (0.17-0.36) | 0.25 (0.17-0.37) | 0.26 (0.17-0.40) |
| Missing or unknown | 0.37 (0.25-0.55) | 0.37 (0.25-0.55) | 0.38 (0.25-0.56) | 0.36 (0.23-0.57) |

^a^Death after COVID was defined as death anytime during study period after first encounter where COVID positive.

**eTables 6-10.** **Odds ratios for the association between race/ethnicity and COVID-19 positivity stratified by comorbidities**

The following tables show the results of a GEE model (binomial distribution, logit link, working independence structure) with robust standard errors and clustering at the neighborhood level. The base model includes listed variables (race/ethnicity, age, sex, CCI), and the fully adjusted model includes all neighborhood factors except those excluded due to multicollinearity.

**eTable 6.** **Odds ratios for the association between race/ethnicity and COVID-19 positivity stratified by obesity status.**

|  | **Not obese** | | **Obese** | |
| --- | --- | --- | --- | --- |
|  | **Base model** | **Full adjusted^a^** | **Base model** | **Full adjusted^a^** |
| Race/Ethnicity |  |  |  |  |
| Non-Hispanic White | 1.00 (referent) | 1.00 (referent) | 1.00 (referent) | 1.00 (referent) |
| Non-Hispanic Black | 4.96 (4.41-5.57) | 3.42 (2.99-3.92) | 3.09 (2.47-3.87) | 2.48 (1.94-3.18) |
| Hispanic/Latino | 7.48 (6.60-8.47) | 4.33 (3.81-4.92) | 4.57 (3.70-5.64) | 3.02 (2.41-3.80) |
| Asian | 2.54 (2.07-3.10) | 1.98 (1.63-2.42) | 4.05 (2.39-6.85) | 3.44 (2.00-5.93) |
| Other | 6.07 (5.23-7.04) | 3.87 (3.36-4.46) | 4.86 (3.58-6.59) | 3.24 (2.4-4.360) |
| Missing or unknown | 4.00 (3.39-4.72) | 3.45 (2.95-4.04) | 2.27 (1.42-3.64) | 1.92 (1.20-3.08) |
| Age (per year increase) | 1.03 (1.03-1.03) | 1.03 (1.03-1.03) | 1.02 (1.02-1.02) | 1.02 (1.02-1.03) |
| Sex |  |  |  |  |
| Female | 1.00 (referent) | 1.00 (referent) | 1.00 (referent) | 1.00 (referent) |
| Male | 1.52 (1.42-1.63) | 1.48 (1.38-1.59) | 1.28 (1.12-1.47) | 1.30 (1.13-1.50) |
| CCI (per unit increase) | 0.95 (0.93-0.98) | 0.95 (0.93-0.97) | 0.98 (0.94-1.02) | 0.97 (0.93-1.02) |

Abbreviations: CCI, Charlson comorbidity index

^a^Includes all neighborhood factors except non-Hispanic White population due to multicollinearity.

**eTable 7.** **Odds ratios for the association between race/ethnicity and COVID-19 positivity stratified by pulmonary disease status.**

|  | **No pulmonary disease** | | **Pulmonary disease** | |
| --- | --- | --- | --- | --- |
|  | **Base model** | **Full adjusted^a^** | **Base model** | **Full adjusted^a^** |
| Race/Ethnicity |  |  |  |  |
| Non-Hispanic White | 1.00 (referent) | 1.00 (referent) | 1.00 (referent) | 1.00 (referent) |
| Non-Hispanic Black | 5.15 (4.56-5.83) | 3.51 (3.06-4.03) | 4.24 (3.65-4.94) | 3.21 (2.68-3.83) |
| Hispanic/Latino | 7.86 (6.85-9.03) | 4.48 (3.90-5.15) | 5.88 (5.06-6.83) | 3.73 (3.20-4.35) |
| Asian | 2.67 (2.14-3.35) | 2.03 (1.62-2.55) | 2.51 (1.84-3.42) | 2.10 (1.53-2.87) |
| Other | 6.18 (5.17-7.39) | 3.80 (3.24-4.46) | 5.92 (4.84-7.25) | 4.09 (3.34-5.01) |
| Missing or unknown | 3.46 (2.86-4.19) | 2.92 (2.43-3.50) | 5.17 (4.09-6.55) | 4.69 (3.73-5.90) |
| Age (per year increase) | 1.03 (1.03-1.03) | 1.03 (1.03-1.03) | 1.03 (1.03-1.03) | 1.03 (1.03-1.04) |
| Sex |  |  |  |  |
| Female | 1.00 (referent) | 1.00 (referent) | 1.00 (referent) | 1.00 (referent) |
| Male | 1.43 (1.31-1.55) | 1.40 (1.29-1.52) | 1.66 (1.52-1.82) | 1.65 (1.51-1.8) |
| CCI (per unit increase) | 0.95 (0.92-0.99) | 0.96 (0.92-0.99) | 0.88 (0.85-0.91) | 0.88 (0.85-0.91) |

Abbreviations: CCI, Charlson comorbidity index

^a^Includes all neighborhood factors except non-Hispanic White population due to multicollinearity.

**eTable 8.** **Odds ratios for the association between race/ethnicity and COVID-19 positivity stratified by chronic kidney disease status.**

|  | **No chronic kidney disease** | | **Chronic kidney disease** | |
| --- | --- | --- | --- | --- |
|  | **Base model** | **Full adjusted^a^** | **Base model** | **Full adjusted^a^** |
| Race/Ethnicity |  |  |  |  |
| Non-Hispanic White | 1.00 (referent) | 1.00 (referent) | 1.00 (referent) | 1.00 (referent) |
| Non-Hispanic Black | 4.74 (4.26-5.27) | 3.28 (2.91-3.71) | 3.46 (2.70-4.43) | 2.87 (2.15-3.85) |
| Hispanic/Latino | 7.40 (6.53-8.38) | 4.27 (3.78-4.82) | 3.78 (2.86-5.00) | 2.64 (1.93-3.61) |
| Asian | 2.57 (2.10-3.14) | 2.06 (1.69-2.50) | 2.30 (1.41-3.74) | 1.78 (1.07-2.97) |
| Other | 6.27 (5.42-7.24) | 3.93 (3.45-4.48) | 3.13 (1.78-5.51) | 2.51 (1.45-4.35) |
| Missing or unknown | 3.78 (3.19-4.48) | 3.25 (2.78-3.81) | 2.99 (1.91-4.67) | 2.70 (1.73-4.22) |
| Age (per year increase) | 1.03 (1.03-1.03) | 1.03 (1.03-1.03) | 1.02 (1.01-1.03) | 1.02 (1.01-1.03) |
| Sex |  |  |  |  |
| Female | 1.00 (referent) | 1.00 (referent) | 1.00 (referent) | 1.00 (referent) |
| Male | 1.48 (1.39-1.58) | 1.45 (1.36-1.55) | 1.32 (1.12-1.57) | 1.35 (1.13-1.61) |
| CCI (per unit increase) | 0.90 (0.87-0.93) | 0.90 (0.87-0.93) | 0.99 (0.94-1.03) | 0.98 (0.94-1.03) |

Abbreviations: CCI, Charlson comorbidity index

^a^Includes all neighborhood factors except non-Hispanic White population due to multicollinearity.

**eTable 9.** **Odds ratios for the association between race/ethnicity and COVID-19 positivity stratified by diabetes mellitus status.**

|  | **No Diabetes Mellitus** | | **Diabetes Mellitus** | |
| --- | --- | --- | --- | --- |
|  | **Base model** | **Full adjusted^a^** | **Base model** | **Full adjusted^a^** |
| Race/Ethnicity |  |  |  |  |
| Non-Hispanic White | 1.00 (referent) | 1.00 (referent) | 1.00 (referent) | 1.00 (referent) |
| Non-Hispanic Black | 4.75 (4.25-5.31) | 3.30 (2.90-3.75) | 3.27 (2.71-3.95) | 2.57 (2.09-3.16) |
| Hispanic/Latino | 7.89 (6.95-8.96) | 4.61 (4.09-5.20) | 3.65 (3.06-4.36) | 2.39 (1.96-2.92) |
| Asian | 2.54 (2.06-3.14) | 2.02 (1.64-2.48) | 2.24 (1.60-3.13) | 1.82 (1.28-2.60) |
| Other | 6.48 (5.59-7.50) | 4.13 (3.61-4.71) | 3.81 (2.87-5.05) | 2.63 (1.98-3.49) |
| Missing or unknown | 3.64 (3.06-4.34) | 3.17 (2.69-3.75) | 4.09 (3.05-5.50) | 3.50 (2.61-4.70) |
| Age (per year increase) | 1.03 (1.03-1.03) | 1.03 (1.03-1.03) | 1.01 (1.01-1.02) | 1.02 (1.01-1.02) |
| Sex |  |  |  |  |
| Female | 1.00 (referent) | 1.00 (referent) | 1.00 (referent) | 1.00 (referent) |
| Male | 1.49 (1.38-1.60) | 1.46 (1.36-1.57) | 1.28 (1.14-1.44) | 1.29 (1.15-1.46) |
| CCI (per unit increase) | 0.95 (0.93-0.98) | 0.95 (0.93-0.98) | 0.94 (0.90-0.97) | 0.93 (0.90-0.97) |

Abbreviations: CCI, Charlson comorbidity index

^a^Includes all neighborhood factors except non-Hispanic White population due to multicollinearity.

**eTable 10.** **Odds ratios for the association between race/ethnicity and COVID-19 positivity stratified by hypertension status.**

|  | **No hypertension** | | **Hypertension** | |
| --- | --- | --- | --- | --- |
|  | **Base model** | **Full adjusted^a^** | **Base model** | **Full adjusted^a^** |
| Race/Ethnicity |  |  |  |  |
| Non-Hispanic White | 1.00 (referent) | 1.00 (referent) | 1.00 (referent) | 1.00 (referent) |
| Non-Hispanic Black | 5.45 (4.77-6.22) | 3.60 (3.09-4.20) | 3.69 (3.20-4.27) | 2.84 (2.41-3.36) |
| Hispanic/Latino | 9.21 (8.00-10.61) | 5.10 (4.44-5.87) | 4.48 (3.86-5.21) | 2.86 (2.41-3.39) |
| Asian | 2.62 (2.06-3.32) | 2.11 (1.67-2.67) | 2.58 (1.98-3.35) | 2.02 (1.53-2.66) |
| Other | 7.28 (6.18-8.58) | 4.41 (3.77-5.16) | 4.30 (3.42-5.41) | 2.97 (2.39-3.70) |
| Missing or unknown | 4.08 (3.37-4.93) | 3.49 (2.91-4.17) | 3.94 (3.10-5.02) | 3.54 (2.8-4.470) |
| Age (per year increase) | 1.04 (1.03-1.04) | 1.04 (1.04-1.04) | 1.02 (1.01-1.02) | 1.02 (1.01-1.02) |
| Sex |  |  |  |  |
| Female | 1.00 (referent) | 1.00 (referent) | 1.00 (referent) | 1.00 (referent) |
| Male | 1.54 (1.42-1.68) | 1.50 (1.38-1.63) | 1.32 (1.21-1.44) | 1.32 (1.21-1.44) |
| CCI (per unit increase) | 0.91 (0.87-0.95) | 0.91 (0.88-0.96) | 1.00 (0.97-1.02) | 0.99 (0.97-1.02) |

Abbreviations: CCI, Charlson comorbidity index

^a^Includes all neighborhood factors except non-Hispanic White population due to multicollinearity.

**Appendix:**

Insurance Status Classification:

Private: Aetna, Allways Health Partners, Blue Cross Blue Shield, BMC Healthnet, Centers Of Excellence, Children’s Medical Security Plan, Cigna, Commonwealth Care Alliance, Coventry First Health, Elder Services, Fallon Health, Generic Commercial, Generic Medicare Replacement, Generic Special Billing, Harvard Pilgrim, Health New England, Hospice, Humana, PHCS Multiplan, Senior Whole Health, Tufts Health Plan, Tufts Health Public Plans, Unicare GIC, United Healthcare

Public: MassHealth, Medicaid Non-Massachusetts, Medicare, Tricare, Veterans Administration, Generic Other Government, ChampVA, Health Safety Net

Other: State Prisons, County Jails, Covid19 HRSA Uninsured Testing and Treatment Fund, Martin's Point, International Commercial, International Embassy, Motor Vehicle, Workers Compensation, Generic Workers' Comp, Informational Self-Pay Claims

Comorbidities from ICD-10 codes in problem list

- Obesity: E66.8, E66.9, E66.2, E66.1, E66.0
- Pulmonary disease: J00-J99
- Chronic kidney disease: N18, I12, I13, E08.22, E09.22, E10.22, E11.22, E13.22
- Diabetes mellitus: E08, E09, E10, E11, E13, E08.22, E09.22, E10.22, E11.22, E13.22, Z79.84, Z79.4
- Hypertension: I10-I16
